# The Veterinary Corps, U. S. Army

**Published:** 1900-01

**Authors:** 


### EDITORIALS.

### THE VETERINARY CORPS, U. S. ARMY.

*"The fearful power of inertia in Washington is worse than any active opposition on any subject. 'Hang on' is the dog that wins."*—Wm. E. Annin.

Such a self-evident fact, as that:

The United States has nearly three million dollars invested in its army horses and mules each year, and its soldiers consume an enormous quantity of animal food annually; and that

The United States is the only civilized government in the world whose army has not a veterinary corps or any proper provision for the care of the health of its animals and for the inspection of the animal food which its soldiers consume; and that

Numerous States have, in the last two decades, established veterinary schools, educating veterinarians to be the scientific peers of other medical men; and that

The work of the Bureau of Animal Industry of the Department of Agriculture, and of the several State Veterinarians, and of the various Agricultural Schools and Experiment Stations, has shown that there are numbers of veterinarians now in this country who, by education, experience, and ability are worthy officials of the government;

Does appeal strongly to everyone who will take the time to consider the subject; but it must be remembered that our legislators in Washington have innumerable important subjects to consider and act upon for the welfare of the government, and that they must choose for immediate action those which have a reason and unquestioned merit.

Fortunately for us, the establishment of a Veterinary Corps in the United States Army equal to that which has been found so valuable and necessary in the armies of all other countries now finds its proper time for immediate legislation.

The very fact that there are certain other needs in the army involving far greater thought and consideration and necessitating the question of vast outlays of money does not in any degree militate against the immediate establishment of an organized Veterinary Service. On the contrary, the service which qualified, competent veterinarians will render to the army will make the more important changes more easily established.

The Spanish War developed the fact that our military condition was inadequate to the conditions of to-day. In thirty-five years, since the interstate war, the population of the country, its commerce, and its standing among other countries had doubled, trebled, quadrupled, while its army had remained at the twenty-five thousand which had been deemed sufficient after the war with Mexico. Now the conditions are vastly different. Our coast line has increased from the Atlantic, Gulf, and Lake borders by the addition of the Pacific States, Alaska, and, last year, the ocean commerce of the Indies, East and West. A half-century ago we imported essentials of life from other countries. To-day we are lowering the trade of England and see before us the competition of Germany and Russia for the supremacy of the civilization of the world.

This means a merchant marine to carry our surplus manufactures and food products to every other country on the earth, a navy to protect our commerce, and a reasonable army for our coast defence and for preserving peace and insuring protection to peaceful citizens where the Stars and Stripes float, and have promised it.

Modern inventions and their appliance to the arts of war have greatly altered the conditions of a half-century ago.

The artillery has grown vastly in importance. Heavy guns, costing each in construction from a few thousand to many thousands of dollars, are needed at each of our ports and in the mobile field artillery, which makes the protection of an army to-day.

Perhaps the greatest need of the army to-day is an Artillery Corps; the expensive guns of our coast defence are rusting and lying idle, while three thousand of our artillerymen are serving as infantry to meet immediate necessities. But, for the efficiency of our field artillery, an ORGANIZED VETERINARY CORPS is needed to insure the purchase of sound horses, to see that they are properly fed and shod, and to care for their health and consequent utility.

The experience of our Indian wars for a quarter of a century and the conditions which face us to-day in Puerto Rico, Cuba, and the Philippines are not those of war, but of the results of war. Public roads must be opened and protected to furnish peaceful travel to the industrious farmer and merchant who develop real civilization. For this purpose armies are not needed, but squads of cavalry which can rapidly patrol the developing country and guard its safety. Over mountain trails pack trains are needed for all transportation, until steam and electricity follow the final civilization.

Ten thousand of our increased army will have to be added to our present ten regiments of cavalry, while the pack trains and quartermasters' trains of army wagons carry supplies to all.

For the efficiency of these, an ORGANIZED VETERINARY SERVICE is a necessity.

While the reorganization of the General Staff in the purely Military Departments of the army is of most urgent necessity, the work of the Quartermaster's Department and of the Commissary Department in the United States army is radically deficient in the absence of an ORGANIZED VETERINARY SERVICE.

The hygienic conditions of animals on transports, the inspection of forage, and the altered rations for animals necessitated by tropical surroundings, the inspection of cattle for food, and of the meat ration for the soldier can only be properly made by competent veterinarians controlled by an organized service.

After all the criticism of the beef furnished to the United States soldiers in the Spanish War, the army has not to-day a qualified meat inspection service. In every other civilized country in the world an ORGANIZED CORPS OF VETERINARIANS inspect the live cattle and the slaughtered meat which the soldiers eat.

The unquestionable standard of the examinations of the Medical Department of the United States Army shows how efficiently a group of talented professional men can be collected who, for money compensation far below that earned in civil life, will devote their best energies to honest labor for the good of their country. A properly organized Veterinary Corps, according rank and recognition, will bring into the service the best element of young, energetic men, whose ambition will be to show that they deserve the place so tardily accorded them.

Under the present system a qualified veterinarian may enter the service with hope, but will not remain in it.

Years ago General Sheridan arranged for the establishment of a Veterinary Service, which his death delayed. To-day General Miles recommends it. Generals Merritt, Brooke, Wilson, Breckenridge, Randolph, and Charles King, General Ludington, the Quartermaster-General, Colonels Lee, Wheeler, Pullman, and other officers of the Quartermaster's Department, and Colonel Hein, Commandant of Cadets at West Point, all have expressed themselves in favor of the establishment of a Veterinary Corps in the army.

The majority of the United States Senators and of the members of Congress have expressed themselves as favorable to the establishment of a Veterinary Corps in the army, and have promised their support to establish it. The President of the United States has expressed himself favorably to it, and yet it will require much labor still to obtain it.

To understand this one must understand what Congress is. Congress is a body of small legislatures. The vast mass of affairs involved in the care of the government are subdivided, and the consideration of them is assigned to committees. On every subject, except those of political party importance or unquestioned merit, the action in regard to them is left to a committee to decide, and the vote in the Senate and the House of Representatives is a perfunctory measure.

Fortunately for us, the ESTABLISHMENT OF A VETERINARY CORPS IN THE ARMY IS ONE OF UNQUESTIONED MERIT, and appeals to the common sense and first instinct of economy in every legislator who has taken the time to consider it. All of the higher authorities in the army have expressed their opinion that its immediate establishment is a necessity.

It does not add an additional cent of cost to the government, for the present expense of incompetent, unorganized civilians by contract is simply to be replaced by an organized service, the competency of which is to be guarded by a proper examination.

The details of soundness in the horses purchased for the government, their shoeing, the quality of their forage, and their hygiene in corrals or in transports, which to-day are left to the accident of the personal interest of the officer in charge, will be given the attention of officers held responsible for them. The meat fed to our soldiers will hereafter be inspected.

*En résumé*, a needed service, which has been neglected in the United States Army, is to be replaced by an organized service such as exists in all the efficient armies in the world except our own.

Senator Kenney, of Delaware, has prepared an amendment for the Army Appropriation Bill, which provides for a small Veterinary Corps, to consist of a Chief Veterinarian, with the rank of Colonel, and thirty Assistant Veterinarians, ten with the rank of First Lieutenant and twenty with the rank of Second Lieutenant. Provision is made for five future promotions—one Veterinarian, with the rank of Major, and four Veterinarians with the rank of Captain—to be competed for as the Corps develops its efficiency. Provision is made for those veterinarians who are now serving in the cavalry regiments, under the act of March 2, 1889, according them rank and permanent positions, with the opportunity of obtaining promotion.

A majority of Congress—the Senate and the House of Representatives—have declared to their constitutents that they favor and will support the legislation for the establishment of this Veterinary Corps; and yet there are many other things which our legislators have to do. Taxes and death are certainties. Meanwhile the soundness of the United States dollar has to be placed beyond controversy; a navy has to be built; agriculture and commerce have to be fostered; the question of Puerto Rico, Cuba, the Philippines, and other matters of vital importance occupy the minds of the President and the statesmen of the country.

A Staff Corps, an Artillery Corps, an increased mobile cavalry, and the necessary transportation for our army, involving hundreds of millions of dollars, requires the attention of the War Department. The details of the few millions invested in the animals of the army seem a small item, though on the improvement of their efficiency lies the foundation of the efficiency of the field artillery, the cavalry, the transportation of the Quartermaster's Department, and the health and safety of our soldiers.

IF THEY THINK, THEY WILL ACT.

This is not a political question; it is one of economy to the government.

We have only to fear INERTIA.

Let every veterinarian, if he has not done so yet, bring the subject before the Governor of his State, agriculturists, horsemen, and the members of his State Legislature, and when they understand the merits of it, and realize the advantages which it will bring, request them to write personal letters to their Senators in Washington, requesting them to bring the subject before the SECRETARY OF WAR and their colleagues in the Senate to SUPPORT SENATOR KENNEY'S AMENDMENT TO THE ARMY APPROPRIATION BILL, ESTABLISHING A VETERINARY CORPS IN THE UNITED STATES ARMY.

